# Treat-to-Target in Axial Spondyloarthritis: Are we there yet?

**DOI:** 10.31138/mjr.33.1.137

**Published:** 2022-04-15

**Authors:** Krystel Aouad, Bassel El-Zorkany

**Affiliations:** 1 Sorbonne University, INSERM, Institut Pierre Louis d’Épidémiologie et de Santé Publique, Paris, France,; 2 Rheumatology Department, Cairo University, Cairo, Egypt

**Keywords:** treat-to-target, management, axial spondyloarthritis

## Abstract

The Treat-to-target (T2T) strategy in axial spondyloarthritis (axSpA) stipulates that treatment should reach a predefined target and if not reached, intensification of therapy is implemented aiming at best future outcomes. Clinical remission was recommended by the 2017 international task force as the treatment target using Ankylosing spondylitis disease activity score (ASDAS) inactive disease, or alternatively, ASDAS low disease activity. The results of a recent T2T trial in axSpA were negative for the chosen primary outcome. Therefore, some concerns are still emerging regarding the optimal treatment target and the weak direct evidence proving the efficacy of such strategy. These challenges among others would preclude the application of T2T strategy in daily clinical practice. This review aims to highlight the updates of the T2T strategy in axSpA, giving an overview of the existing treatment targets, their potential benefits, and challenges to apply this strategy.

## INTRODUCTION

The treat-to-target (T2T) strategy is a growing paradigm in chronic diseases stipulating that treatment should aim at a prespecified target. The T2T concept relies on defining a specific target that should be highly correlated to future irreversible outcomes. It should also include evidence that better outcomes will develop if this target is maintained below a precise cut-off value. As opposed to usual care, this treatment target should be reached in a well-defined period and be sustained over time. If not achieved, this T2T approach allows escalating treatment to attain this goal.

In the view of preventing irreversible outcomes, the T2T strategy has gained importance in chronic conditions such as diabetes and hypertension^1,2^ and is now being also applied to rheumatic diseases such as rheumatoid arthritis,^3^ gout,^4^ and psoriatic arthritis (PsA).^5^ Although there is sparse direct evidence on its long-term benefits, this T2T strategy was also recommended in axial spondyloarthritis (axSpA) in 2014.^6,7^ This arises from indirect evidence that inhibition of the inflammatory process in axSpA and better control of disease activity is associated with inhibition of future structural damage causing physical impairment.^6,8^ However, challenges remain regarding this strategy and the choice of the optimal target to achieve. The objective of this review is to discuss the updates of the T2T strategy in axSpA, give an overview of the predefined treatment targets and their correlation to structural damage with a highlight on the current evidence and controversies of such strategy that would preclude its application.

## THE OPTIMAL TREATMENT TARGETS

Treat-to-target strategy relies primarily on defining the specific target. Identifying the ideal treatment target in axSpA is a crucial step that may be challenging because of disease heterogeneity that includes axial and peripheral symptoms as well as extra-musculoskeletal manifestations (EMM). The target in chronic inflammatory diseases, particularly in axSpA and PsA, is to achieve remission/inactive disease.^6^ Clinical remission is defined as the absence of clinical and laboratory evidence of significant disease activity: of both musculoskeletal involvement (arthritis, dactylitis, enthesitis, axial disease) and EMM.^6^ Once remission is achieved, this state should be sustained over time. It is usually recommended to monitor disease activity at least 6 monthly^9^ or even closer (e.g., every 1–3 months) in patients with high disease activity.^6^

In clinical trials, different disease activity composite scores have been used to define remission (**Table 1**); most commonly: the Assessment of SpondyloArthritis international Society partial remission criterion (ASAS-PR), the Ankylosing spondylitis disease activity score (ASDAS) inactive disease (ID), or low Bath ankylosing spondylitis disease activity index (BASDAI) scores.^10^ The ASAS-PR criterion is determined by a score less than 2 on a 0 to 10 scale in each of the four domains: axial pain, inflammation (morning stiffness), patient global assessment (PGA) and function.^11^ The BASDAI includes 5 patient-reported outcomes (PROs) based on pain (axial, peripheral, entheseal), morning stiffness and fatigue, whereas ASDAS integrates both PROs (axial and peripheral pain, morning stiffness, PGA) and objective measures of inflammation (CRP or ESR).^10^ ASDAS has a possible advantage compared to other composite scores: first, t he ASASPR includes the physical function domain which is less sensitive to change in advanced axSpA; second, the ASDAS is a highly discriminatory tool and incorporates validated cut-offs for remission and low disease activity which is not the case for BASDAI.^10,12^ Therefore, a potential target in axial SpA should be precisely determined using a well-discriminatory tool (eg, ASDAS).

**Table 1:** Composite scores defining remission: potential targets of the treat-to-target strategy in axial spondyloarthritis.

**Disease activity composite scores defining remission**	**Target to reach**	**Time to reach target**	**Components of each composite score**							
			**Axial pain**	**Peripheral pain**	**Morning stiffness**	**Entheseal pain**	**Fatigue**	**Patient global**	**Physical function**	**ESR or CRP**
**ASDAS inactive disease**	<1.3 or <2.1	<6 months	X	X	X			X		X
**BASDAI**	No validated cut-offs for remission*	<6 months	X	X	X	X	X			
**ASAS-PR**	<2/10 on each domain	<6 months	X		X			X	X	

* *There are no validated cut-offs for remission with BASDAI; the cut-offs used in trials are below 4, 3 or 2 or % reduction. ASDAS: Ankylosing Spondylitis Disease Activity Score; ASAS-PR: Assessment of SpondyloArthritis international Society partial remission criteria; BASDAI: Bath Ankylosing Spondylitis Disease Activity Index; CRP: C-reactive protein; ESR: Erythrocyte Sedimentation Rate.*

Given the importance to reach remission, the first T2T recommendations in spondyloarthritis (axSpA and PsA) were initiated in 2014^7^ and were subsequently updated in 2017 by the international T2T task force.^6^ They both advocate that the treatment target should aim to reach inactive disease/clinical remission or alternatively, low disease activity (LDA).^6^ In 2017, the ASDAS inactive disease (ID) with a score below 1.3 was recommended as the preferred outcome measure to define remission.^6^ If not possible to reach, LDA with a score between 1.3 to 2.1 may be an acceptable alternative target.

Besides clinical remission, disease activity can objectively be observed through disappearance of MRI inflammatory lesions that tend to decrease considerably in patients with clear clinical response.^13^ It is acknowledged that the presence of inflammation on MRI is associated with structural progression.^13^ However, whether imaging remission -mainly MRI remission- should be a treatment target, is not yet recommended and remains of research interest.^13,14^ Overall, ASDAS-ID has been proposed as the T2T target to define remission, however, many concerns related to the optimal treatment target remain open to debate and require further validation in clinical practice.

## THE EVIDENCE BEHIND A SUCCESSFUL TREAT-TO-TARGET APPROACH

The T2T recommendations have been updated in 2017, even though there are still sparse direct data validating the real benefits of such a T2T approach in comparison to usual care. However, the recommendations were based on robust indirect evidence supporting the potential success of this approach to prevent structural damage in axSpA.

Early diagnosis of axSpA, better understanding of disease pathogenesis with emergence of new therapeutic agents have reformed the management era of axSpA, offering better clinical outcomes and therapeutic ways to prevent irreversible damage. It is clear today that there is apparently an association between disease activity and structural progression in axSpA.^8,15,16^ Therefore, early abrogation of inflammation is considered a potential way to inhibit radiographic progression. In the 12 years OASIS longitudinal study, an increase in one unit of the ASDAS, engendered an increase in radiographic progression by 0.7 on the modified Stoke Ankylosing Spondylitis Spine Score (mSASSS).^17^ Interestingly, higher radiographic inhibition was seen in patients with ASDAS-ID compared to patients with very high disease activity (>3.5) or even with LDA.^17^

Additionally, biologic disease-modifying anti-rheumatic drugs (bDMARDs), both tumor necrosis factor (TNF) inhibitors and interleukin (IL)-17 inhibitors approved in axSpA, have shown evidence of delaying structural progression in axSpA.^8,18,19^ This finding is in line with the potent effect of bDMARDs on the inhibition of inflammatory pathways in axSpA.^8,20^

Increasing evidence shows that in PsA and axSpA, tight control of disease activity may inhibit structural progression, still, the best evidence emerges from trials comparing a T2T approach to usual care (UC). In axSpA, the TICOSPA trial is the only trial published to date.^21^ This trial did not demonstrate the superiority of the T2T strategy (target ASDAS <2.1) compared to UC for the primary outcome chosen: the ASAS-Health Index (ASAS-HI). Although not statistically significant, the percentage of patients achieving ≥ 30% improvement on the ASAS-HI was numerically higher in T2T group (47.3%) compared to UC (36.1%) after 1 year. As for the secondary outcomes, ASAS-ID was not statistically different between the 2 arms, however, ASAS-LDA and ASAS40 were superior in the T2T arm (76.5% and 52.3%) compared to UC (59.5% and 34.7%, p<0.05 respectively). Interestingly, adverse events were not increased in the T2T arm despite a higher prescription rate of biologics. There were also positive outcomes from a societal economic perspective: 0.04 additional QALY in the T2T arm.^21^

Some results of TICOSPA study that were not statistically significant m ight have happened due to study limitations. The main limitation was related to the choice of the primary outcome measure: the ASAS-HI that would seem inappropriate to evaluate the treatment efficacy between both groups in the context of a T2T strategy. The ASAS-HI can assess the global level of functioning and health and would not directly measure disease activity. Instead, it would reflect consequences of having an active disease.^22^ Other limitations were related to the methodology and design of the study. A higher-than-expected response rate was seen in UC. Centers included in the study were SpA expert centers who would apply T2T-like strategy as their UC. Additionally, the post-hoc power calculation was very low (ß=29.9%) and the sample size was small (80 patients/arm) compared to the post-hoc sample size calculation that indicated the need of 440 patients per arm for a power of 80% in a cluster randomised design.^23^

Other trials on T2T are ongoing in axSpA. The STRIKE trial (NCT02897115) was terminated because of slow recruitment. The TReat-to-tArget with seCukinumab in Axial Spondyloarthritis study (TRACE, NCT03639740) is an ongoing trial and has defined A SDAS-ID as the treatment target with an imaging outcome as the primary endpoint, that is, the proportion of patients with a positive change in MRI-inflammation of the spine and sacroiliac joints between week 16 and 24. The AScalate trial (NCT03906136) is another ongoing T2T study with secukinumab defining the treatment target as the ASDAS clinically important improvement (change from baseline ≥ 1.1) and if not achieved at week 12, an increase in treatment dose was planned. The ASAS40 at week 24 was set as the primary endpoint in this study.^24^

Overall, a T2T strategy may seem beneficial i n the management of axSpA, even though relying on limited evidence and a recent negative trial.

## CHALLENGES OF T2T STRATEGY

Although the T2T concept seems very promising in axSpA, many challenges remain and are open to debate. First, concerning disease activity scores, the use of ASDAS alone to define remission is not universally accepted in such a heterogeneous disease. The ideal target for disease activity would be a composite index that includes the different clinical manifestations, objective measures of systemic inflammation, PROs for quality of life and physical function as well as structural progression scores.^25^ Yet, there is no composite score today that incorporates all these domains. In clinical practice, rheumatologists take into account other aspects of the disease to define a patient in remission.^26,27^ Both NSAIDs use and EMM (eg., inflammatory bowel disease (IBD), psoriasis and uveitis) not included in ASDAS, ASAS-PR and BASDAI, are important elements to consider when assessing a patient with a good clinical response under treatment.^26^ For instance, low ASDAS scores do not rule out uveitis or IBD flares, instead, the appearance of such EMM under treatment can lead to treatment change irrespective to ASDAS scores.

Furthermore, the treatment target should be simple enough to be applied in clinical practice while not very stringent to achieve.^28^ Nevertheless, recent evidence has shown that ASDAS inactive disease is very stringent and only a small proportion of patients are able to achieve.^29,30^ Data from the DESIR cohort showed that only 17% out of 614 patients achieved ASDAS-ID after 5 years of follow-up.^30^ This constitutes a real barrier to the T2T strategy since ASDAS-ID was recently recommended as the outcome measure to use.

In a T2T strategy for axSpA, it is important to reach the target but also to define t he t ime t o reach t he target as well as the minimal duration of sustained remission required to inhibit structural progression. Although not based on any evidence, it is usually considered that treatment target should be reached within 6 months.^7^

Moreover, the ASAS/European Alliance for Associations for Rheumatology (EULAR) has proposed at least 6 months of sustained remission before tapering of bDMARDs.^31^ This recommendation was also based on expert opinion.^31^ Therefore, there is still an unmet need for further exploring these points which are an integral part of the T2T strategy.

Finally, this T2T strategy aiming at ASDAS <1.3 or <2.1, was not endorsed by the recent 2019 American College of Rheumatology/Spondylitis Association of America/Spondyloarthritis Research and Treatment Network (ACR/SAA/SPARTAN).^32^ They recommended against the use of T2T approach over usual care. The reason behind this was the lack of strong direct evidence confirming its benefits and the risk of rapid cycling across all available treatments to reach the target.^32^

## CONCLUSION

Overall, the T2T strategy which aims to reach the ASDAS-ID or alternatively ASDAS-LDA, constitutes a promising approach in patients with axSpA. However, there is still a lack of direct strong evidence to support such strategy with a recent negative trial. Furthermore, challenges remain concerning the optimal treatment target to use that would incorporate all aspects of the disease, the time to reach the target and the duration of sustained remission to achieve the improved future outcomes. A better understanding of disease predictors of remission is needed to guide the choice of the optimal treatment target. More data are still awaited to confirm the efficacy of such T2T strategy on long-term outcomes compared to UC. Finally, despite many achievements in that context, we are not there yet.
